# THE IMPACT OF LEISURE ACTIVITIES ON OLDER ADULTS’ COGNITIVE FUNCTION

**DOI:** 10.1093/geroni/igad104.3117

**Published:** 2023-12-21

**Authors:** Jungjoo Lee, Seokmin Oh, Miriam Rodriguez, Junhyoung Kim

**Affiliations:** University of Southern Mississippi, Hattiesburg, Mississippi, United States; Indiana University Bloomington, Bloomington, Indiana, United States; Indiana University Bloomington, Bloomington, Indiana, United States; Indiana University Bloomington, Bloomington, Indiana, United States

## Abstract

The purpose of the present study was to investigate the relationship between types of leisure activities (i.e., Leisure-Time Physical Activity (LTPA), intellectually stimulating activities, and community-based activity and the cognitive functions of older adults. We extracted 3,767 samples from the 2020 Health and Retirement Study (n = 15,723). Cognitive functions: (a) memory was assessed by both immediate and delayed recall tests, b) working memory was measured by a subtraction-by-7 test, and (c) attention and processing speed was assessed by the counting back word test. Hierarchical regression analysis was used to investigate the relationship between types of leisure and cognitive functions. LTPA, intellectual leisure, and community-based leisure were significantly associated with improved memory function. The intellectual activity was significantly associated with improved working memory and improved attention and processing speed. This finding provides practical implications for healthcare providers and therapists to design and implement various types of leisure for older adults.

